# Novel green soybean shuidouchi fermented by *Bacillus velezensis* with multibioactivities

**DOI:** 10.1002/fsn3.2579

**Published:** 2021-10-02

**Authors:** Hong Liu, Shen Luo, Jun Liu, Qiaojuan Yan, Shaoqing Yang, Zhengqiang Jiang

**Affiliations:** ^1^ Key Laboratory of Food Bioengineering (China National Light Industry) College of Engineering China Agricultural University Beijing China; ^2^ Beijing Advanced Innovation Center for Food Nutrition and Human Health College of Food Science & Nutritional Engineering China Agricultural University Beijing China

**Keywords:** antioxidant activity, *Bacillus velezensis*, fibrinolytic activity, green soybean, multibioactivity, shuidouchi

## Abstract

Soybeans are usually fermented by *Bacillus subtilis* to produce *shuidouchi*, which is a traditional fermentation soybean product in China. In the study, green soybeans were fermented by *Bacillus velezensis* to make a novel green soybean shuidouchi with multibioactivities. The processing conditions were optimized as follows: initial moisture content 75%, inoculum concentration 7 log CFU/g, and incubation time 24 h for prefermentation; water addition 50%, salt addition 6%, temperature 45°C, 3 days for postfermentation. The fermented green soybean shuidouchi (FGSS) showed 234.8 FU/g dry weight (DW) for the fibrinolytic activity and IC_50_ of 0.33 mg/ml for the anticoagulant activity. FGSS had higher contents of chemical components including 3.6 mg rutin (RE)/g DW of total flavonoids, 8.2 mg gallic acid (GAE)/g DW of total phenolics, 63.7 mg/g DW of reducing sugars, and 163.8 mg/g DW of peptides than the unfermented green soybean shuidouchi (UGSS). Moreover, it exhibited high antioxidant activities of 29.8, 85.1 μmol trolox equivalent (TE)/g DW, and 12.8 μmol Fe^2+^/g DW through 2,2‐diphenyl‐1‐picrylhydrazyl (DPPH), 2,2’‐azino‐bis (3‐ethylbenzthiazoline‐6‐sulfonic acid) diammonium salt (ABTS), and ferric reducing antioxidant power (FRAP) experiments. Thus, a novel green soybean shuidouchi fermented by *B. velezensis* owing to multibioactivities can provide a theoretical basis for the further development of functional shuidouchi.

## Graphical abstract

In the study, green soybeans were fermented by Bacillus velezensis to make a novel green soybean Shuidouchi with multi‐bioactivities. The fermented green soybean Shuidouchi (FGSS) showed 234.8 FU/g dry weight (DW) for the fibrinolytic activity and IC50 of 0.33 mg/ml for the anticoagulant activity.

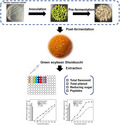

## 1 INTRODUCTION

*Douchi*, a traditional soybean food in China, has often been consumed as delicious condiment with functional effects (Wang et al., 2021). *Shuidouchi* is a typical bacteria‐fermented douchi, which has a history of seasoning or dish for thousands of years. The product is in a wet state with a high water content (Luo et al., 2010). The fermentation process of shuidouchi includes two stages: (1) soaked and cooked soybeans are prefermented by microorganisms until viscin threads produced among soybeans, and (2) sterile water and some auxiliary materials such as salt, pepper powder, and other species are added to the prefermented soybeans, which are further postfermented for 1 ~ 2 weeks (Chen, Liu, et al., 2019b). Shuidouchi contains many functional components including polypeptides, oligosaccharides, isoflavones, saponins, and other microbial secondary metabolites. The health benefits of shuidouchi includes protecting the intestinal tract and preventing colitis (Zhang et al., 2019), preventing constipation in mice (Chen, Zhang, et al., 2019a), antioxidant activities (Xu et al., 2015), and antimutagenic and anticancer effects (Zhang et al., 2019). Furthermore, shuidouchi has been found to possess a certain concentration of fibrinolytic enzyme, which may have a thrombolytic effect to prevent thrombus (Chen, Liu, et al., 2019b).

*Bacillus* species is the main microorganisms present in shuidouchi and plays an important role in the fermentation processes. Both *Bacillus subtilis* and *Bacillus amyloliquefaciens* have been used as good starters to produce shuidouchi (Chen, Liu, et al., 2019b). So far, soybean is the raw material of shuidouchi, and its variety affects both sensory characteristics and physicochemical properties of products (Luo et al., 2010). The contents of protein, moisture, and ash in green soybeans are similar to those of soybeans. However, the carbohydrate content of green soybeans is 4 times higher than that of soybeans, and the lipid content is very low. Green soybeans with the contents of high carbohydrate and low fat may be more suitable for the processing material of shuidouchi (Feng et al., 2015). *Bacillus velezensis*, as a novel species, was recently taxonomically classified as a closely related species *B. amyloliquefaciens* (Ye et al., 2018). So far, this species has been widely separated from diverse habitats, such as soil, water, and fermented foods. *B. velezensis* is potential starter culture of fermented foods (Cho et al., 2018). Recently, *B. velezensis* BS2 from a traditional Korean fermented seafood and *B. velezensis* SW5 from fish sauce showed fibrinolytic activity (Yang et al., 2020; Yao et al., 2019). A novel fermented soybean food by various microbes (*B. subtilis* GD 1, *B. subtilis* N4, *B. velezensis* GZ1, *L. bulgaricus*, and *H. anomala* CICC 1728) showed peptide, total phenol, total flavonoid contents, suitable organic acid and biological amine contents, strong antioxidant and antifatigue activities (Cui et al., 2020). Liu et al. (2020) reported that *B. velezensis* DP‐2 isolated from douchi was applied in soybean meal fermentation, it degrades soybean antigen proteins and improved crude protein, soluble protein and trichloroacetic acid–soluble protein to make it more suitable for animal feed. So far, no information is available regarding the soybean fermented food by single strain *B. velezensis*. There is no study on the green soybeans fermented by *B. velezensis* to make shuidouchi.

A strain showing high fibrinolytic activity, *B. velezensis* CAU263, was newly isolated from Chinese traditional fermented douchi. In this study, the strain was used to ferment green soybean shuidouchi (FGSS). The processing conditions were optimized, and the potential nutrients and multibioactivities of green soybean shuidouchi were further investigated. This is the first report on shuidouchi fermented by *B. velezensis*.

## 2 MATERIALS AND METHODS

### 2.1 Materials, microorganism, and reagents

Green soybeans were obtained from a local farm (Harbin, China). Salt, paprika, and ginger powder were purchased from a supermarket (Beijing, China). *B. velezensis* CAU263 (CGMCC NO: 20318) isolated from Chinese traditional douchi was used in this study. Fibrinogen from bovine plasma, thrombin, DPPH, ABTS, angiotensin I‐converting enzyme (ACE), 2,4,6‐tris (2‐pyridyl)‐s‐triazine (TPTZ), (±)‐6‐hydroxy‐2,5,7,8‐tetramethyl‐chromane‐2‐carboxylic acid (trolox), 3‐tert‐butyl‐4‐hydroxyanisole (BHA), and hippuryl‐L‐histidyl‐L‐leucine (HHL) were obtained from Sigma‐Aldrich Co. Ltd. Peptone and yeast extract were obtained from Oxoid Co. Ltd. Rutin was gained from Shanghai Yuanye Biotechnology Co. Ltd. Gallic acid was obtained from Shanghai Macklin Biochemical Co. Ltd. L (+)‐ascorbic acid (VC) was acquired from Fujifilm Wako Pure Chemical Corporation. Other chemical reagents were obtained from Beijing Chemical Works.

### 2.2 Preparation of green soybean shuidouchi

*Bacillus velezensis* CAU263 was cultivated at 37°C for 8 h in LB medium (200 rpm) and used as a starter culture. The green soybeans (100 g dry weight) were first washed and soaked in distilled water (300 ml) for 10 ~ 18 h at 25°C. The soaked green soybeans were steamed at 121°C for 20 min and cooled to room temperature for prefermentation.

Prefermentation was performed by inoculating 5% (culture/total material, ν/ω) starter culture (10^7^ CFU/g) into the substrate. The prefermented green soybeans were stored in a refrigerator (4°C, 24 h) for maturation after incubation at 37°C for 24 h. For postfermentation, the prefermented green soybeans were mixed with 50% (water/total material, ν/ω) sterilized water which contained 5% salt, 3% paprika, and 3% ginger powder. Green soybean shuidouchi was finally produced after incubation at 45°C for 1 ~ 7 days (Figure. S1). All samples were stored at −20°C after freeze‐drying, pulverizing, and passing through a 20‐mesh sieve.

### 2.3 Optimization of process conditions of green soybean shuidouchi

For optimization of prefermentation, the initial moisture content (60%, 65%, 70%, 75%, 80%), inoculum concentration (3, 4, 5, 6, 7 log CFU/g), and incubation time (12, 16, 20, 24, 28 h) were investigated by the single factor method. The orthogonal test which included three factors and three levels was further used to optimize conditions.

For further optimization of postfermentation, the water addition (50%, 65%, 80%, 95%, 110%), salt concentration (2%, 4%, 6%, 8%, 10%), postfermentation temperature (25, 30, 35, 40, 45, 50°C), and postfermentation time (1, 2, 3, 4, 5, 6, 7 days) were carried out using the one‐factor‐at‐a‐time method.

The sensory properties of green soybean shuidouchi were evaluated (Lan et al., 2020). For all sensory evaluation, rating scores of 1 ~ 25 were used as follows: the scores of 21 ~ 25 were excellent, the scores of 16 ~ 20 were good, the scores of 11 ~ 15 were moderate, the scores of 6 ~ 10 were poor, and the scores of 1 ~ 5 were inferior. The sensory evaluation score was the sum of color, appearance, flavor, and taste scores.

The sensory evaluation score and amino acid nitrogen content were used as evaluation indexes of green soybean shuidouchi to calculate their weighted average score as a comprehensive score for optimization in the following Equation (1). A higher comprehensive score indicated better quality (Dong et al., 2020).

(1) $$Comprehensivescore=\frac{\mathrm{Sensoryscoreofthesample}}{\mathrm{Maximumsensoryscoreofallsamples}}*100*0.7+\frac{A\min oacidnitrogencontentofthesample}{Maximuma\min oacidnitrogencontentofallsamples}*100*0.3$$

### 2.4 Extraction methods of green soybean shuidouchi

Water extract and ethanol extract were prepared. A 10‐fold volume of distilled water was added to the shuidouchi freeze‐dried powder and extracted using a shaking incubator at 200 rpm (25°C, 2 h). After centrifugation at 11,510 *g* for 10 min, the supernatant was collected and stored at 4°C for analysis. For ethanol extract, a 4‐fold volume of 80% ethanol was added to the shuidouchi freeze‐dried powder and extracted 20 min by using ultrasonic (480 W). After centrifugation at 1,840 *g* for 10 min, the supernatant was collected. Furthermore, a 3‐fold volume of 80% ethanol was mixed with the sediment, and the previous extraction step was repeated two times. The supernatant was combined as ethanol extract and stored at 4°C for analysis.

### 2.5 Determination of amino acid nitrogen

The amino acid nitrogen content was determined by the Chinese national standard (GB 5009.235‐2016). In short, 5 g powder samples were added to 100 ml deionized water to homogenize, and the supernatant was obtained after centrifugation at 2,880 *g* for 10 min. The supernatant 10 ml was titrated to pH 8.2 with 50 mM NaOH. Acidity was calculated using the consumed volume of titrant. Amino was then fixed by adding excess formaldehyde to the solution. The samples were titrated to pH 9.2 with 50 mM NaOH afterward. The amino acid nitrogen content was determined using the consumed volume of titrant.

### 2.6 Determination of peptides and ACE inhibiting activity

The content of peptides was estimated by the o‐phthaldialdehyde (OPA) spectrophotometric assay. OPA solution contained 25 ml of 100 mM sodium tetrahydroborate, 10 ml of 5% sodium dodecyl sulfate (SDS), 1 ml of 40 mg/ml OPA (dissolved in methanol), and 0.1 ml of β‐mercaptoethanol. The volume was adjusted to 50 ml with deionized water. The reaction was performed by adding 10 μl water extract solution to 400 μl OPA solution for 8 min at room temperature, and the absorbance was recorded at 340 nm. The content of peptides was represented as mg/g DW by using Gly–Leu as the standard.

Angiotensin I‐converting enzyme inhibiting activity was determined by using the fluorescence‐based method (Hong et al., 2016); the IC_50_ value was used to express the result of ACE‐inhibiting activity, which is defined as the green soybean shuidouchi concentration (mg/mL) required to inhibit 50% ACE activity.

### 2.7 Determination of total phenolic contents (TPCs)

The total phenolic contents (TPCs) were measured using the modified Folin–Ciocalteu reagent method (Shi et al., 2020), namely, 80 μl water extract solution was loaded on a 96‐well microplate, and 80 μl Folin–Ciocalteu reagent (50%) and 80 μl of 10% sodium carbonate solution were added and mixed well. After incubation in the dark at 30°C for 30 min, the absorbance at 760 nm was recorded. TPC samples were represented as mg GAE/g DW based on a standard curve of gallic acid (GAE).

### 2.8 Determination of total flavonoid contents (TFCs)

The total flavonoid contents (TFCs) were estimated using the aluminum chloride colorimetric assay with slight modification (Li et al., 2015). In short, ethanol extract solution (100 μl) was mixed with 6 μl of 5% NaNO_2_ in a 96‐well microplate and reacted for 6 min. After 6 μl of 10% AlCl_3_ was added to react for additional 6 min, the reaction was terminated by 80 μl of 4% NaOH. Finally, the total volume was adjusted to 200 μl with 50% ethanol. After 15 min, the absorbance at 510 nm was read. The calibration curve was made using rutin (RE) as a standard. TFC of samples were represented as mg RE/g DW.

### 2.9 Determination of reducing sugars

The contents of reducing sugars were estimated using the dinitrosalicylic acid (DNS) method (Miller, 1959), namely, 3 ml water extract solution was added to a 10‐mL test tube and mixed with 3 ml DNS reagent. The test tubes were immediately cooled to ambient temperature using distilled water after reaction at 100°C for 10 min and measured at 540 nm. The contents of reducing sugars were expressed as mg/g DW calculated by the calibration curve used glucose as a standard.

### 2.10 Determination of fibrinolytic activity

The fibrinolytic activity of water extract solution was estimated by Huy et al. (2016). In short, 0.4 ml of 0.72% fibrinogen solution was mixed with 1.4 ml of 50 mM phosphate buffer (pH 8.5) in test tubes and kept at 37°C for 5 min. The fibrin clot was formed by adding 0.1 ml of 20 U/ml thrombin solution and left at 37°C for 10 min. Then 0.1 ml water extract solution was added, and the reaction was conducted at 37°C for 60 min with stirring every 20 min. In the end, 2 ml of 0.2 mol trichloroacetic acid (TCA) was added to terminate the reaction. The absorbance was monitored at 275 nm after centrifugation at 11,510 *g* for 10 min. In the assay, one unit of fibrinolytic activity (FU) was defined as the amount of enzyme making 0.01 increase in absorbance in 1 min.

### 2.11 Determination of anticoagulant activity

The anticoagulant activity was evaluated using the previous method with minor modification (Wei et al., 2011), namely, 50 mM Tris–HCl buffer (pH 7.2) was used as the solvent for fibrinogen and thrombin. Both 140 μl of 0.1% fibrinogen solution and 40 μl water extract solution were mixed in 96‐well microplates. Then, the reaction was performed by adding 10 μl of 12 U/ml thrombin solution to the 96‐well plates for 10 min at 37°C and measured at 405 nm. The IC_50_ value was used to express the result of anticoagulant activity which was defined as green soybean shuidouchi concentration (mg/ml) required to inhibit 50% anticoagulant activity.

### 2.12 Determination of antioxidant activity

The assay of DPPH radical scavenging capacity was spectrophotometrically estimated by Shi et al. (2020) with minor modification. In brief, 200 μl methanolic solution of DPPH (0.1 mM) was mixed with 40 μl water extract solution in a 96‐well microplate and then measured at 517 nm after storage for 30 min in the dark. The antioxidant activity against ABTS^+^ was evaluated using the modified spectrophotometric assay by Sancho et al. (2015). The reactions were performed in a 96‐well microplate at room temperature for 6 min after adding 10 μl water extract solution to 190 μl ABTS^+^ solution and then the absorbance values were read at 734 nm. Trolox was used as a reference. The results were represented as μmol trolox equivalent (TE) per gram lyophilized powder of shuidouchi (μmol TE/g DW). FRAP activity was determined according to the previous report (Li et al., 2015). The FRAP values were represented as μmol Fe^2+^ per gram lyophilized powder of shuidouchi (μmol Fe^2+^/g DW).

### 2.13 Statistical analysis

All results were performed in triplicate. Statistical analysis was carried out using SPSS 19.0 software (SPSS, USA) and expressed as mean value ± standard deviation. Statistical significance was determined at *p* < 0.05.

## 3 RESULTS AND DISCUSSION

### 3.1 Effects of prefermentation conditions on FGSS quality

**FIGURE 1:**
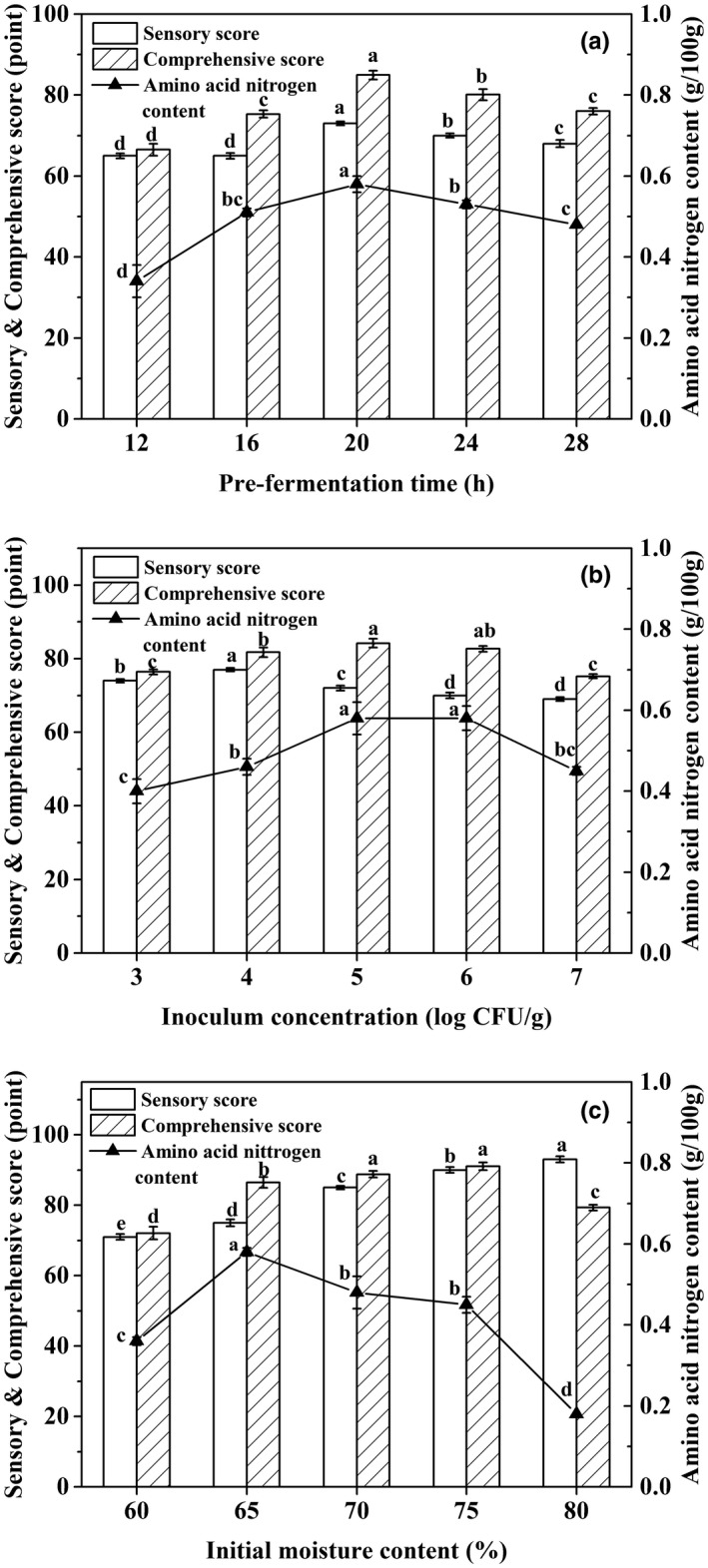
Effects of prefermentation conditions including prefermentation time (a), inoculum concentration (b), and initial moisture content (c) on FGSS quality. The prefermentation time, inoculum concentration, and initial moisture content of FGSS were investigated by the single factor method. Data represent mean ± standard deviation (*n* = 3). Different letters in the same color indicate significantly different values (*p* < 0.05)

The effects of prefermentation conditions on FGSS quality are shown in Figure 1. The sensory score and amino acid nitrogen content of FGSS increased from 65.0 to 73.0 and from 0.34 g/100 g to 0.58 g/100 g with the prefermentation time from 12 to 20 h, respectively. The comprehensive score reached the highest of 85.0 at 20 h (Figure 1a). The inoculum concentration exhibited a slight influence on the sensory score and amino acid nitrogen content of FGSS. The highest comprehensive score was 84.2 at 5 log CFU/g of inoculum concentration (Figure 1b). With the increment of initial moisture content, the sensory score increased from 71.0 to 93.0 and the amino acid nitrogen content was decreased. The highest comprehensive score of 91.0 was obtained at 75% of initial moisture content (Figure 1c).

Furthermore, the effects of prefermentation conditions on FGSS quality using the orthogonal array are shown in Table S1 of the Supplementary material. According to the magnitude order of *R*, the effect of prefermentation factors on FGSS quality decreased in the following order: prefermentation time, inoculum concentration, and initial moisture content. The optimized combination was A_2_B_3_C_3_ according to the magnitude order of K, namely, 75% initial moisture content, 7 log CFU/g inoculum concentration, and 24 h prefermentation time. Under this condition (A_2_B_3_C_3_), the sensory score and amino acid nitrogen content were 90.0 and 0.41 g/100g, respectively. Moreover, the comprehensive score was up to 90.1 (Table S1).

The quality of fermented soybean products during the processing conditions are usually related to many factors such as the fermentation microorganism, inoculum concentration, substrate moisture, fermentation temperature, and time (Shahzad et al., 2020). In the production of shuidouchi, increment in the initial moisture content can alter the nutrient solubility and scavenge wastes of metabolites in the substrate which promote the growth of microorganisms (Rodriguez de Olmos & Selva Garro, 2020). Excessive moisture content causes reduction in oxygen transfer and heat dissipation. The inoculum concentration, fermentation temperature, and time were found to be important factors in the production of FGSS (Table S1). With cell growth, the fibrinolytic enzyme was biosynthesized, and the yield was growth‐associated with *B. subtilis* (Wei et al., 2011). More bacteria may lead to a decrease in the amount of nutrition of per cell in a certain weight substrate, or even be damaged or dead because of oxygen deficiency and the accumulation of metabolic wastes (Feng et al., 2015). In addition, appropriate incubation temperature and time were needed for the growth and physiological processes of microorganisms. It has been reported that microorganisms may synthesize only a reduced number of proteins essential for growth and other physiological processes at high temperature. Longer incubation period and higher incubation temperature may also lead to the accumulation of toxic inhibitors and inhibition of metabolite production and enzyme activity (Rodriguez de Olmos & Selva Garro, 2020).

### 3.2 Effects of postfermentation conditions on FGSS quality

**FIGURE 2:**
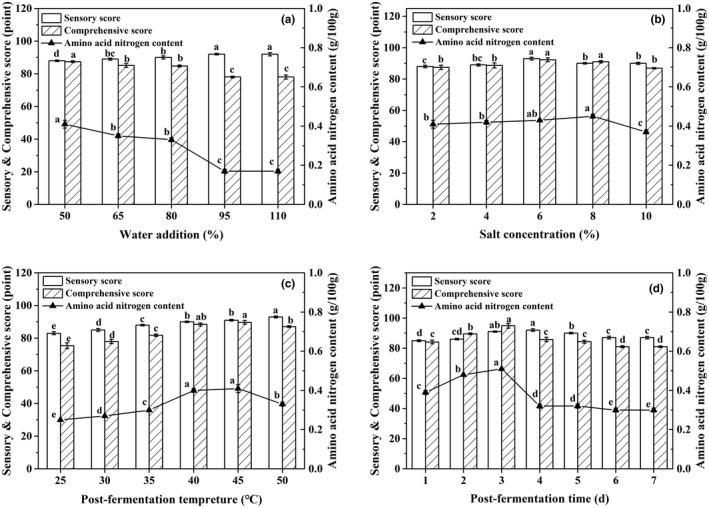
Effects of postfermentation conditions including water addition (a), salt concentration (b), fermentation temperature (c), and fermentation time (d) on FGSS quality. The water addition, salt concentration, postfermentation temperature, and postfermentation time of FGSS were optimized using the one‐factor‐at‐a‐time method. Data represent mean ± standard deviation (*n* = 3). Different letters in the same color indicate significantly different values (*p* < 0.05)

The effects of postfermentation conditions on FGSS quality are shown in Figure 2. With the addition of water from 50% to 110%, the sensory score was continuously improved from 88.0 to 92.0 (Figure 2a). After the addition of various salt concentrations, the contents of amino acid nitrogen in FGSS ranged from 0.37 to 0.45 g/100 g. The sensory score of FGSS increased from 88.0 to 93.0 when the salt concentration increased from 2% to 6% and then decreased (Figure 2b). As the postfermentation temperature rose from 30°C to 40°C, the contents of amino acid nitrogen increased. At 45°C, FGSS contained 0.41 g/100 g of amino acid nitrogen with 91.0 of sensory score (Figure 2c). The content of amino acid nitrogen was the highest (0.51 g/100 g) after postfermentation for 3 days. The sensory score was 91.0 and then decreased after postfermentation for 4 days (Figure 2d). Overall, FGSS showed the highest comprehensive score of 94.9 under the optimized conditions.

To further enhance the quality of shuidouchi, the process of ripening (postfermentation) is important. Postfermentation can largely contribute to not only the improved bioactivities but also the form of characteristic flavor and taste (Tan et al., 2019). FGSS was prefermented for 24 h and then postfermented for 3 days in the study (Figures 1 and 2). The total fermentation time of FGSS was 4 days, obviously shorter than that of traditional douchi which usually ferments from several weeks to months. Addition of NaCl to fermented soybean products can improve the flavor and taste as well as inhibit harmful microorganisms for extending shelf life (Kim et al., 2020). The salt concentration in the FGSS product was almost 3% (Figure 2), which is lower than that of traditional fermented douchi. In the preparation of *doenjang*, higher concentration of NaCl caused reduction in isoflavone aglycones, especially daidzein and genistein which have greater anti‐inflammatory and bioavailability effects than other forms of isoflavones (Kim et al., 2018). FGSS can be considered a low‐salt fermented soybean food, which may fit the demand of public nutritional health.

The relationship between amino acid nitrogen content and the sensory of shuidouchi was also explored. Amino acid nitrogen is considered as the metabolite of microorganism of proteins that can reflect the degree of aging of douchi (Zhang et al., 2021). As an important index of douchi products, its content is related to the flavor (Lan et al., 2020). The amino acid nitrogen content of FGSS was 0.51 g/100 g (Figure 2) which met the Sichuan provincial standard of douchi (DB 51 T391‐2006) stating that the amount of amino acid nitrogen of shuidouchi should be ≥0.2 g/100 g. In the previous report, the mean amino acid nitrogen content of shuidouchi samples was 0.39 g/100 g (Chen, Liu, et al., 2019). Usually, traditional douchi is fermented by various microorganisms in the air. The natural fermentation process may cause some problems such as the long period of fermentation, unstable quality, and potential safety risks (Tan et al., 2019). The use of pure starter (*B. velezensis* CAU263) in the study had been demonstrated to improve the quality of FGSS and shorten the fermentation process.

### 3.3 Chemical components of FGSS

The chemical components of FGSS including the total flavonoids, total phenolics, reducing sugars, and peptides were analyzed (Table 1). Compared with the UGSS, TFC of FGSS was improved by 19.5% from 2.89 mg RE/g DW to 3.59 mg RE/g DW. TPCs and the content of reducing sugars in FGSS were 4.5 and 3.0 times higher than those of UGSS (1.82 → 8.16 mg GAE/g DW, 21.4 → 63.7 mg/g DW). Specifically, a significant increment was detected on the content of peptides, which increased from 13.9 mg/g DW in UGSS to 163.8 mg/g DW in FGSS.

**TABLE 1:** Chemical components contents of UGSS and FGSS under the optimal conditions

Samples	Total flavonoid (mg RE/g DW)	Total phenol (mg GAE/g DW)	Reducing sugar (mg/g DW)	Peptides (mg/g DW)
UGSS	2.89 ± 0.07^b^	1.82 ± 0.04^b^	21.40 ± 0.02^b^	13.92 ± 0.14^b^
FGSS	3.59 ± 0.09^a^	8.16 ± 0.62^a^	63.66 ± 0.24^a^	163.82 ± 3.59^a^

Note

Data represent mean ± standard deviation (*n* = 3). ^a,b^ Different letters in the same column indicate significantly different values (*p* < 0.05).

Abbreviations: DW, dry weight; FGSS, fermented green soybean shuidouchi; UGSS, unfermented green soybean shuidouchi.

Apart from the flavor improvements, the significant increment in chemical components in FGSS had been found. Total phenolic contents in FGSS were improved compared with UGSS (Table 1). The correlation analysis indicated that polyphenols could be the major contributors of antioxidant activities (Khosravi & Razavi, 2021). Recently, many researchers have reported on the enhanced antioxidant activities with the increased phenolic and flavonoids contents of fermented soybeans (Li et al., 2020; Shahzad et al., 2020). Pigeon peas fermented by *B. subtilis* improved the total flavonoids (2.5 → 5.4 mg/g) and total phenolic contents (10.8 → 15.2 mg/g) during fermentation (Lee et al., 2015); 181.5% increment of the TPC in soybean fermented by *B. amyloliquefaciens* was reported in another study (Shahzad et al., 2020). Therefore, FGSS may be used as a good functional food source which is rich in chemical components.

### 3.4 ACE inhibitory activity of FGSS

**FIGURE 3:**
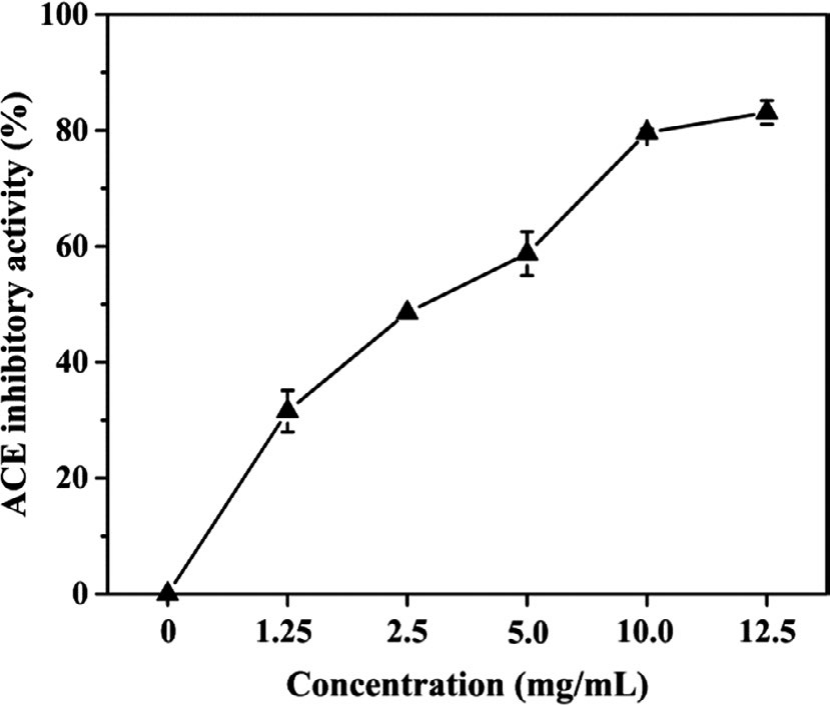
ACE inhibitory activity of FGSS. The ACE inhibitory activity of FGSS using different sample concentrations. Data represent the mean ± standard deviation (*n* = 3)

Angiotensin I‐converting enzyme inhibitory activity of FGSS is shown in Figure 3. The ACE inhibitory activity reached 80%, and the IC_50_ of FGSS was 0.82 mg/ml at 10 mg/ml of FGSS, whereas the UGSS did not have ACE inhibitory activity. This result indicated that the high ACE inhibitory activity of FGSS should be mainly produced during the fermentation by *B. velezensis* CAU263. The ACE inhibitory activities of some traditional fermented soybean foods are much different. The IC_50_ values of *tofuyo*, *soy sauce*, *miso paste*, *douchiqu*, and *sufu* were 1.77 mg/ml, 3.44 mg/ml, 1.27 mg/ml, 5.09 mg/ml, and 0.79 ~ 1.31 mg/ml, respectively (Li et al., 2016). Furthermore, ACE inhibitory activity of 76.5% was obtained after ripening for 2 weeks of douchi sample (Wang et al., 2016). Compared with these studies, FGSS may be considered as an outstanding source of ACE inhibitors based on the strong ACE inhibitory activity.

### 3.5 Thrombolytic activities of FGSS

**FIGURE 4:**
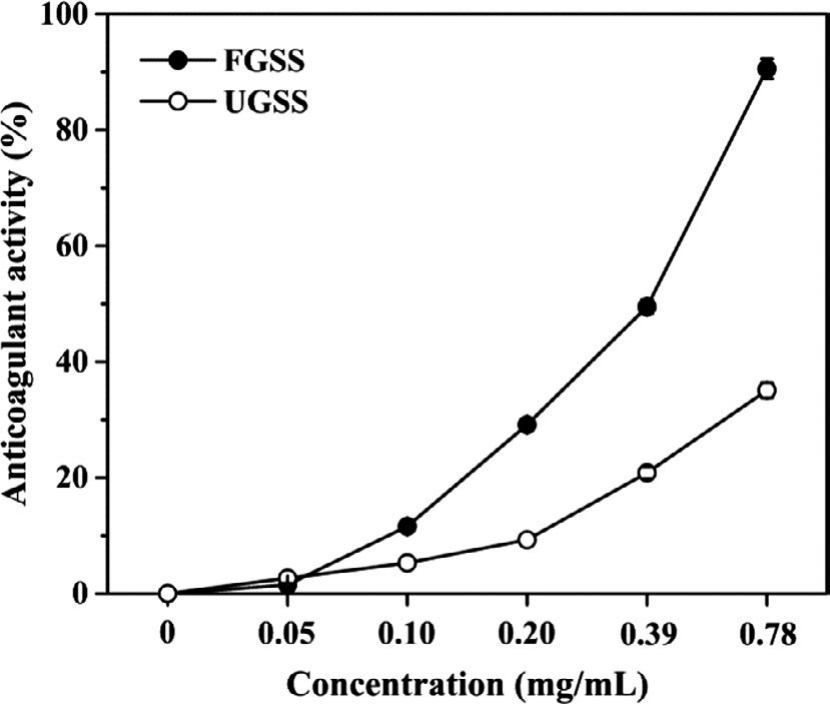
Anticoagulant activity of UGSS (O) and FGSS (●). The anticoagulant activity of UGSS and FGSS using different sample concentrations. Data represent mean ± standard deviation (*n* = 3)

The anticoagulant activity of FGSS showed a dose‐dependent manner (Figure 4). FGSS exhibited the highest anticoagulant activity of 90.6%, with the IC_50_ of 0.33 mg/ml at 0.78 mg/ml of FGSS, much higher than that of UGSS (35.1%), with the IC_50_ of 1.14 mg/ml. Moreover, the fibrinolytic activity of FGSS was 234.8 FU/g DW, whereas the UGSS did not have fibrinolytic activity.

IC_50_ of the anticoagulant activity in FGSS was 0.33 mg/ml, which is 3.5 times lower than that of UGSS (1.14 mg/ml) (Figure 4). Moreover, FGSS showed high fibrinolytic activity of 234.8 FU/g DW. The fibrinolytic activities of fermented soybean oil cake powder produced by four isolated strains *B. amyloliquefaciens* CB1, *B. amyloliquefaciens* CB2, *B. amyloliquefaciens* MB1, and *B. amyloliquefaciens* MB2 were in a range from 29.7 to 77.9 FU/g (Huy et al., 2016). The fibrinolytic activity of 48.8 FU/g was gained according to the fermentation of pigeon pea by *B. subtilis* 14,715 (Lee et al., 2015). Moreover, the fibrinolytic activity of chickpeas fermented by *B. amyloliquefaciens* was up to 39.28 FU/g (Wei et al., 2011). FGSS using *B. velezensis* CAU263 in this study had very high fibrinolytic activity. These suggest that FGSS has great potential to be developed as functional foods with high fibrinolytic activities.

### 3.6 Antioxidant activities of FGSS

**FIGURE 5:**
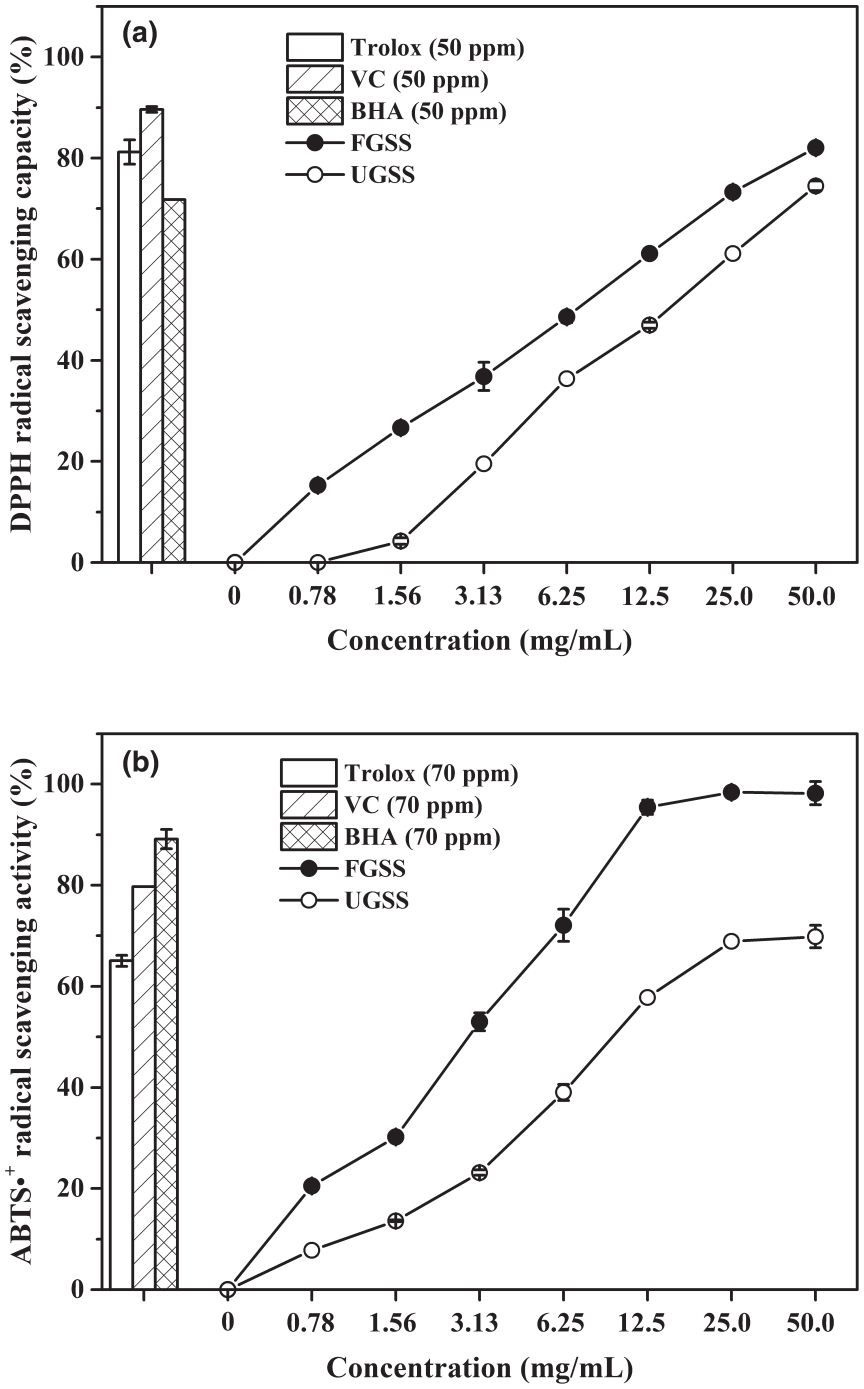
DPPH radical scavenging capacity (a) and ABTS•^+^ radical scavenging activity (b) of UGSS (O) and FGSS (●). The DPPH radical scavenging capacity of UGSS and FGSS using different sample concentrations. The ABTS radical scavenging activity of UGSS and FGSS using different sample concentrations. Data represent mean ± standard deviation (*n* = 3)

The antioxidant activities of FGSS are shown in Figure 5. The DPPH radical scavenging activities of UGSS and FGSS increased from 4.3% to 74.5% and 15.3% to 82.0% with the increment of their concentrations (1.56 ~ 50.0 mg/ml, 0.78 ~ 50.0 mg/ml) (Figure 5a). The IC_50_ of FGSS was 6.6 mg/ml, and IC_50_ of UGSS was 14.9 mg/ml. The DPPH values of FGSS and UGSS was 29.8 and 11.8 μmol TE/g DW, respectively. Furthermore, the DPPH values of FGSS was 2.5 times higher than those of UGSS. Li et al. (2020) reported that the enhancement of potential antioxidants of fermented soybean was improved in DPPH radical scavenging activity by 61.1%. Lee et al. (2019) found that DPPH radical scavenging activity was improved by 11.9%~87.0% of fermented soybeans. The DPPH radical scavenging activity (75.2%) was observed after fermentation in the mixed culture of *B. subtilis* GD1, *B. subtilis* N4, and *B. velezensis* GZ1 (Cui et al., 2020). The maximum ABTS^+^ radical scavenging activity reached 98% at 25.0 mg/ml of FGSS (Figure 5b). The IC_50_ of FGSS was 2.6 mg/ml and IC_50_ of UGSS was 10.1 mg/ml. The ABTS^+^ values were improved from 40.5 μmol TE/g DW in UGSS to 85.1 μmol TE/g DW in FGSS. ABTS•^+^ radical scavenging activity was enhanced by 86% after soybeans fermented by *B. subtilis* (Ali et al., 2018). Also, ABTS•^+^ radical scavenging activity increased from 7.62% to 81.08%, with increment of fermented soybean meal concentration from 0.5 to 8 mg/ml (Dai et al., 2017). Moreover, FGSS had a higher ferric reducing antioxidant power (12.8 μmol Fe^2+^/g DW) than that of UGSS (6.1 μmol Fe^2+^/g DW).

Several studies had emphasized the importance of the fermentation process owing to the production of antioxidant compounds (Salim et al., 2017). The potential antioxidants in FGSS may be phenolics, flavonoids, and peptides, which explained the high antioxidant activities during fermentation. Similarly, fermented red beans showed an improvement of DPPH radical scavenging activity, total phenols, and total flavonoids during fermentation (Li et al., 2020). The fermented tofu could also promote the transformation of phenolics with low antioxidant activity into their corresponding forms with high antioxidant activity (Yin et al., 2020). The increased DPPH radical scavenging activity and FRAP values have a significant positive correlation between total phenols and total flavonoids in *cheonggukjang* (Piao & Eun, 2020). The microorganisms may play a vital role in the antioxidant activities of fermented foods. *Mucor*‐ and *Aspergillus*‐fermented douchi had higher DPPH value (2.7 ~ 23.1 μmol TE/g) than the bacteria‐fermented *Natto* (2.6 ~ 4.5 μmol TE/g). FRAP assay showed significant difference between douchi (9.1 ~ 11.6 μmol Fe^2+^/g) and *natto* (4.7 ~ 6.1 μmol Fe^2+^/g) (Xu et al., 2015). In the current study, FGSS using *B. velezensis* CAU263 exhibited the high antioxidants.

## 4 CONCLUSION

A novel green soybean shuidouchi using *B. velezensis* CAU263 as a starter was developed. The optimal process conditions for green soybean shuidouchi improved the contents of total phenols, total flavonoids, peptides, and reducing sugars. The green soybean shuidouchi also had high fibrinolytic enzyme and antioxidant activities. The study may be helpful for the development of shuidouchi with multibioactivities, which has the potential to improve human health.

## CONFLICTS OF INTEREST

The authors declare that there are no conflicts of interest.

## ETHICAL APPROVAL

This study does not involve any human or animal testing.

## Supporting information

Supplementary Material

## Data Availability

The data that support the findings of this study are available from the corresponding author upon reasonable request.
