# Supplementary material for: Novel green soybean shuidouchi fermented by Bacillus velezensis with multibioactivities
Source: Food Sci Nutr. 2021 Oct 2;9(12):6538–47. doi: 10.1002/fsn3.2579 (PMC8645744; doi:10.1002/fsn3.2579)
Supplement: Supplementary file 1 — Supplementary Material [file FSN3-9-6538-s001.docx]

Supplementary material

**Novel green soybean *Shuidouchi* fermented by *Bacillus velezensis* with multi-bioactivities**

**Hong Liu^1^, Shen Luo^2^, Jun Liu^2^, Qiaojuan Yan^1^, Shaoqing Yang^2^, Zhengqiang Jiang^2,*^**

*^1^ Key Laboratory of Food Bioengineering (China National Light Industry), College of Engineering, China Agricultural University, Beijing 100083, China*

*^2^ Beijing Advanced Innovation Center for Food Nutrition and Human Health,* *College of Food Science & Nutritional Engineering, China Agricultural University, Beijing 100083, China*

**Corresponding authors. Tel.: +86 10 62737689; fax: +86 10 82388508. E-mail: zhqjiang@cau.edu.cn (Z. Q. Jiang).*

**Table S1** Results of L_9_ (3^3^) orthogonal experiment of pre-fermentation of FGSS.

| Test number | A | B | C | Sensory score (point) | Amino acid nitrogen content (g/100 g) | | Comprehensive score (point) | |
| --- | --- | --- | --- | --- | --- | --- | --- | --- |
| 1 | 70 | 5 | 20 | 83.5±0.5^e^ | 0.26±0.03^e^ | | 75.86±1.9^d^ | |
| 2 | 80 | 5 | 16 | 94.0±0.8^a^ | 0.10±0.02^f^ | | 75.26±0.2^d^ | |
| 3 | 70 | 7 | 24 | 88.0±1.0^bc^ | 0.57±0.00^a^ | | 95.50±0.4^a^ | |
| 4 | 75 | 3 | 20 | 75.0±0.5^f^ | 0.45±0.04^b^ | | 79.53±1.7^c^ | |
| 5 | 80 | 7 | 20 | 86.0±0.9^d^ | 0.40±0.00^c^ | | 85.10±0.3^b^ | |
| 6 | 75 | 5 | 24 | 89.0±0.7^b^ | 0.33±0.00^d^ | | 83.64±1.3^b^ | |
| 7 | 70 | 3 | 16 | 87.0±2.0^cd^ | 0.12±0.03^f^ | | 71.10±0.6^e^ | |
| 8 | 80 | 3 | 24 | 87.0±0.5^cd^ | 0.31±0.02^d^ | | 81.10±1.7^c^ | |
| 9 | 75 | 7 | 16 | 89.0±1.5^b^ | 0.26±0.04^e^ | | 79.96±0.8^c^ | |
| k1 | 80.82 | 77.24 | 75.44 |  |  | |  | |
| k2 | 81.04 | 78.25 | 80.16 |  |  | |  | |
| k3 | 80.49 | 86.85 | 86.75 |  |  | |  | |
| R | 0.55 | 9.61 | 11.31 |  |  | |  | |
| Optimal levels | A_2_ | B_3_ | C_3_ |  | |  | |  |
| Important order | C>B>A |  |  |  | |  | |  |

Data represent the mean ± standard deviation (n=3); FGSS, fermented green soybean *Shuidouchi*. A: initial moisture content (%); B: inoculum concentration (log CFU/g); C: post-fermentation time (h). Different letters in the same column indicate significantly different values(*p*<0.05).


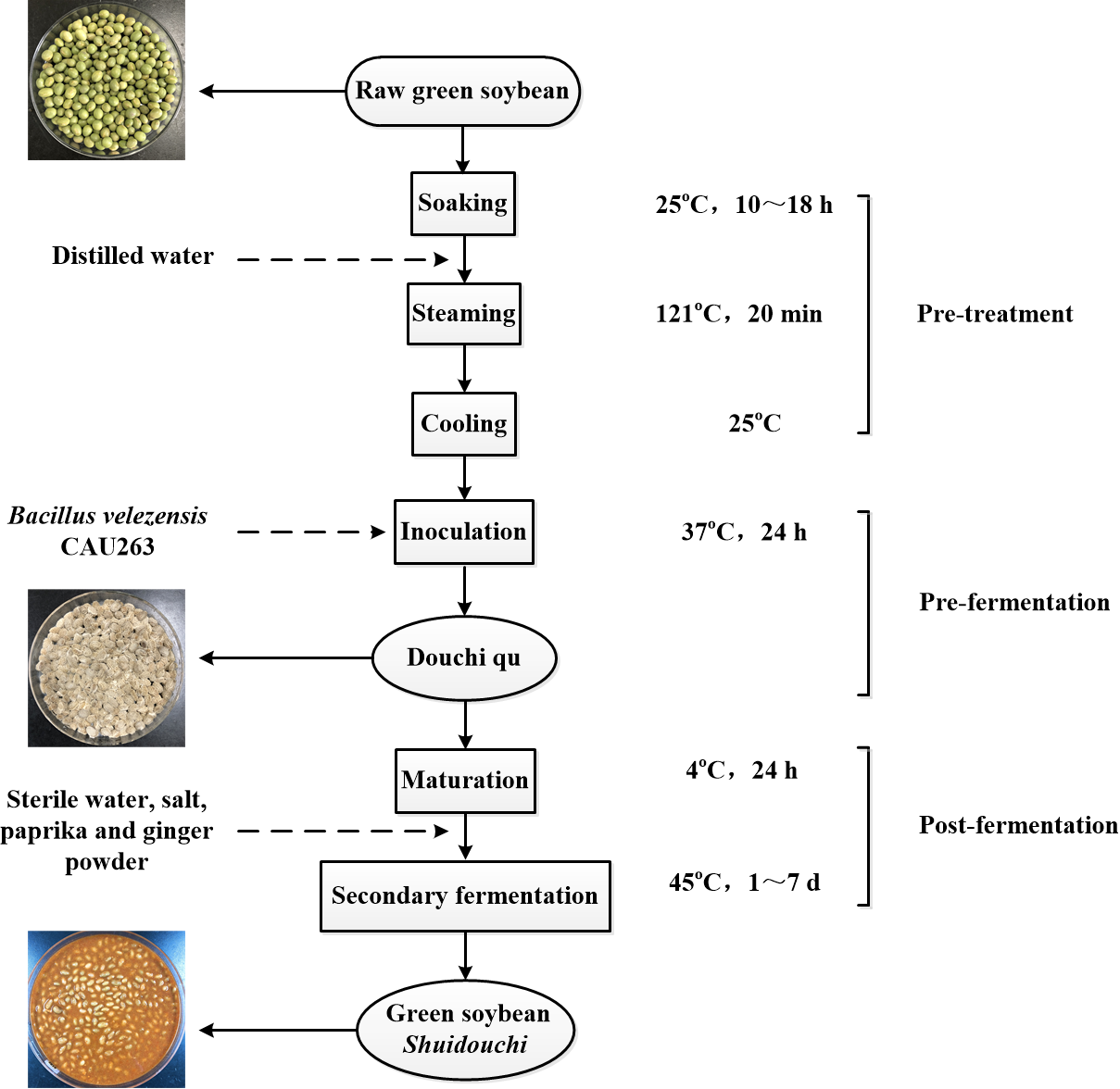


**Fig. S1** Flow diagram for processing of green soybean *Shuidouchi*.
